# NMR Dynamic View of the Destabilization of WW4 Domain by Chaotropic GdmCl and NaSCN

**DOI:** 10.3390/ijms25137344

**Published:** 2024-07-04

**Authors:** Liang-Zhong Lim, Jianxing Song

**Affiliations:** Department of Biological Sciences, Faculty of Science, National University of Singapore, 10 Kent Ridge Crescent, Singapore 119260, Singapore

**Keywords:** Hofmeister series, chaotropic ion, protein denaturation, protein conformation, protein stability, protein dynamics, NMR spectroscopy

## Abstract

GdmCl and NaSCN are two strong chaotropic salts commonly used in protein folding and stability studies, but their microscopic mechanisms remain enigmatic. Here, by CD and NMR, we investigated their effects on conformations, stability, binding and backbone dynamics on ps-ns and µs-ms time scales of a 39-residue but well-folded WW4 domain at salt concentrations ≤200 mM. Up to 200 mM, both denaturants did not alter the tertiary packing of WW4, but GdmCl exerted more severe destabilization than NaSCN. Intriguingly, GdmCl had only weak binding to amide protons, while NaSCN showed extensive binding to both hydrophobic side chains and amide protons. Neither denaturant significantly affected the overall ps-ns backbone dynamics, but they distinctively altered µs-ms backbone dynamics. This study unveils that GdmCl and NaSCN destabilize a protein before the global unfolding occurs with differential binding properties and µs-ms backbone dynamics, implying the absence of a simple correlation between thermodynamic stability and backbone dynamics of WW4 at both ps-ns and µs-ms time scales.

## 1. Introduction

Many proteins must adopt specific three-dimensional structures to function in cells [1]. The thermodynamic stability of a protein refers to the free energy difference between its folded and unfolded states, relying on the balance of noncovalent interactions within the protein and with its surroundings [2,3,4]. On the other hand, proteins also possess inherent dynamics, involving structural motions and fluctuations across various time scales [5,6,7]. It is now well recognized that the biological functions of proteins and their interactions with other molecules, as well as their aggregation which is accountable for diverse diseases, are not only governed by structures and thermodynamic stability, but also modulated by protein dynamics [2,3,4,5,6,7]. Indeed, the study of protein folding, thermodynamic stability and dynamics has attracted the attention of protein chemists for more than 100 years [1,2,3,4,5,6,7]. However, despite exhaustive studies, the relationship between thermodynamic stability and the dynamics of proteins remains not fully understood [2,3,4,5,6,7].

**Figure 1:**
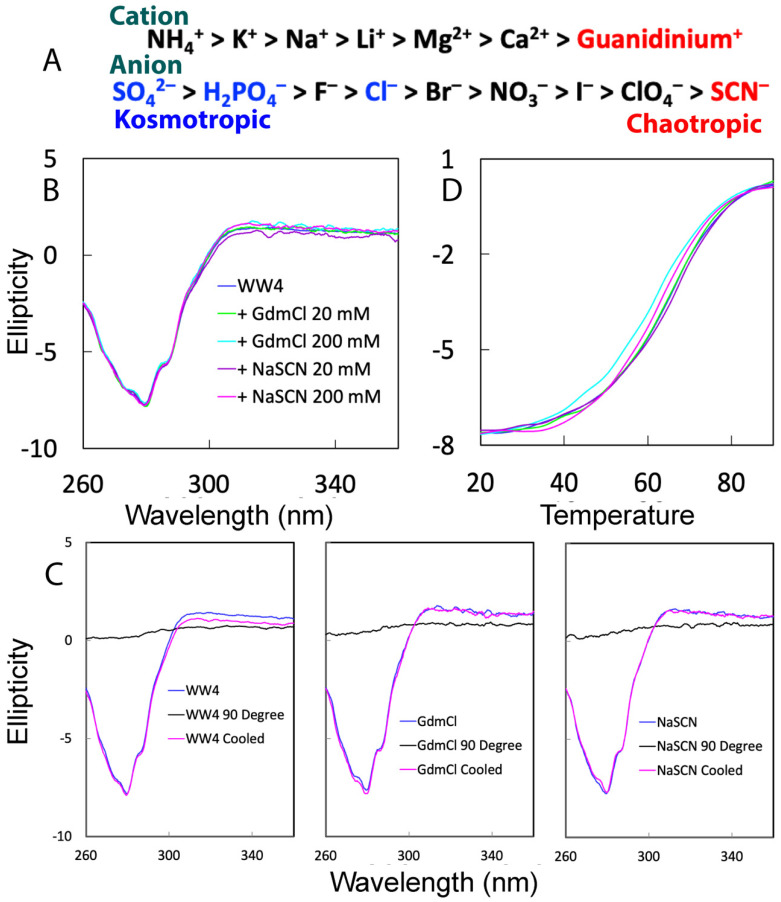
CD characterization of the tertiary packing and thermal stability. (**A**) Hofmeister series of common cations and anions. (**B**) Near-UV CD spectra of the WW4 domain under five different conditions. (**C**) Near-UV CD spectra of WW4 at 25 °C and 90 °C as well as at 25 °C, cooled down after the thermal unfolding in the absence and in the presence of GdmCl and NaSCN at 200 mM. (**D**) Thermal unfolding curves of ellipticity at 280 nm under five different conditions.

One common method to experimentally measure protein thermodynamic stability is to denature a protein with chemical denaturants such as salt ions. The Hofmeister series is a classification of ions based on their ability to affect the stability of proteins in water [4,8,9,10]. Ions that increase the stability of proteins are called kosmotropes, while ions that decrease the stability of proteins are called chaotropes. The Hofmeister series ranks the ions according to their kosmotropic or chaotropic effects, in which the cation guanidinium and the anion thiocyanate are two examples of strong chaotropic ions (Figure 1A). Guanidinium chloride (GdmCl) and sodium thiocyanate (NaSCN) are two strong chemical denaturants which have been widely employed to measure protein thermodynamic stability. However, despite extensive investigations with experimental and computational approaches, the microscopic mechanisms by which chemical denaturants including GdmCl and NaSCN destabilize a protein remain largely controversial [10,11,12,13,14,15,16,17,18,19,20].

Recently, we have attempted to explore the mechanisms by which five salts including Na_2_SO_4_, Na_2_HPO_4_, NaCl, GdmCl and NaSCN affect the conformations, thermal stability, binding and backbone dynamics of a 39-residue but well-folded WW4 domain of a ubiquitin ligase WWP1 over a range of low-salt concentrations (≤200 mM) by use of CD spectroscopy to assess its tertiary packing and thermal stability, as well as NMR spectroscopy to characterize its conformations, binding and backbone dynamics across ps-ns and μs-ms time scales. Previously, we have determined the NMR structure of WW4 (2OP7) to adopt the classic WW fold, and also found no detectable self-association even at a concentration up to 2 mM [21]. Most advantageously, unlike a large and folded protein whose hydrophobic side chains are buried in the core and thus inaccessible to solvent molecules in the native state, WW4 is only composed of a flat three-stranded β-sheet whose hydrophilic and hydrophobic residues appear to be largely accessible in the native state. In this context, even before WW4 becomes globally unfolded, most of the WW4 residues should be available to interact with denaturant molecules even at low concentrations. Consequently, the binding occurrences, if any, should be detectable by NMR 1D and HSQC spectroscopy, which are powerful in detecting binding and perturbation events with residue-specific resolution, encompassing affinities spanning from very high to very low, even when the dissociation constant (Kd) is in the millimolar range [21,22,23,24,25].

Here, we presented the results with two denaturants GdmCl and NaSCN, which indicate that up to 200 mM, both denaturants exhibited no detectable effect on the tertiary packing of WW4. Nevertheless, GdmCl was able to destabilize WW4 more severely than NaSCN. Unexpectedly, up to 200 mM GdmCl showed no detectable binding to the hydrophobic side chains of WW4, while NaSCN displayed extensive binding. Intriguingly, although GdmCl and NaSCN exerted no significant effects on the backbone dynamics of WW4 on ps-ns time scales, they distinctively affected the backbone dynamics of WW4 on μs-ms time scales. Our CD and NMR study provides residue-specific insights into the denaturant-induced alterations of the conformations, stability, binding and backbone dynamics of the WW4 domain, implying that even for the chaotropic salts GdmCl and NaSCN, they appear to destabilize WW4 by distinctively interacting with WW4 residues and altering dynamic properties on the µs-ms time scale.

## 2. Results

### 2.1. CD Characterization of Tertiary Packing and Thermal Stability of WW4

Previously, we have collected and analyzed the far-UV CD spectra of the 39-residue WW4, whose solution structure was determined by NMR [21]. WW4 has a far-UV CD spectrum typical of a β-strand-rich protein [21]. However, here because the presence of GdmCl or NaSCN would generate very high non-specific noise over the far-UV CD region, we decided to assess the effects of two denaturants on the tertiary packing and thermal stability of WW4 by using the near-UV CD region, which reports the protein tertiary packing. Briefly, we have acquired near-UV CD spectra of WW4 under five different conditions, namely: without any denaturant and with GdmCl or NaSCN at 20 mM and 200 mM, respectively (Figure 1B). The presence of the negative signal of near-UV spectra at 280 nm indicates that despite being very small, WW4 does have a tight tertiary packing. Furthermore, very similar near-UV CD spectra in the absence and presence of GdmCl or NaSCN strongly indicate that WW4 has no significant alterations in the tertiary packing at all five conditions.

We subsequently determined the thermodynamic stability under five conditions by performing the thermal unfolding measurements on WW4 without any Cys residue, as we previously conducted on the FUS RRM domain also containing no Cys [26]. Figure 1C displays the CD spectra of WW4 at 25 °C and 90 °C in the absence and presence of GdmCl and NaSCN at 200 mM, along with the spectra of the samples cooled down to 25 °C after thermal unfolding. The results indicate: (1) at 90 °C, the tertiary packing of WW4 was eliminated under these conditions; (2) however, when three unfolded samples were cooled down to 25 °C, WW4 became refolded with the native tertiary packing completely restored; and (3) the unfolding processes of the WW4 domain in the absence and in the presence of GdmCl and NaSCN up to 200 mM are all reversible. This is fundamentally different from what was previously observed on the FUS RRM domain, which underwent dynamic self-association upon being unfolded and thus had an irreversible thermal unfolding process [26].

Furthermore, Figure 1D presents the unfolding curves, namely the changes in ellipticity at 280 nm upon gradually increasing the temperature from 20 to 90 °C for the WW4 domain in the absence and in the presence of GdmCl or NaSCN at 20 mM and 200 mM. Noticeably, despite its small size of only 39 residues and the absence of any disulfide bridge, WW4 exhibits a remarkably high melting temperature (Tm) of 63.5 °C. This Tm is even higher than that of many larger folded proteins, including the 87-residue RRM domain of FUS with a Tm of ~52 °C [26], and the 140-residue human profilin 1 (hPFN1) with a Tm of ~56 °C [27]. On the other hand, the WW4 samples in the presence of GdmCl or NaSCN at 20 mM have thermal unfolding curves which are very similar to that without any denaturants, indicating no detectable destabilization of WW4 in the presence of two denaturants at 20 mM. Nevertheless, on the other hand, both denaturants at 200 mM did reduce the thermal stability. Specifically, the melting temperature of WW4 without denaturant is ~63.5 °C, which reduced to ~54.5 and ~60.3 °C, respectively, for samples with 200 mM GdmCl and NaSCN (Figure 1D). The results clearly indicated that GdmCl decreased the melting temperature by 9 °C, and NaSCN only by 3.2 °C. As such, GdmCl is a much stronger denaturant than NaSCN, completely consistent with the classic ranking [3,8,9,10].

### 2.2. NMR Characterization of the Binding/Perturbation

Subsequently, we assessed the binding of two denaturants with WW4 as monitored by 1D and HSQC NMR spectroscopy, which offers significant advantages for characterizing protein–ligand interactions. It provides high-resolution structural information, allowing precise identification of binding sites and conformational changes upon ligand binding, as well as determination of dissociation constants spanning from very high to very low, even when the dissociation constant (Kd) is in the millimolar range [21,22,23,24,25].

**Figure 2:**
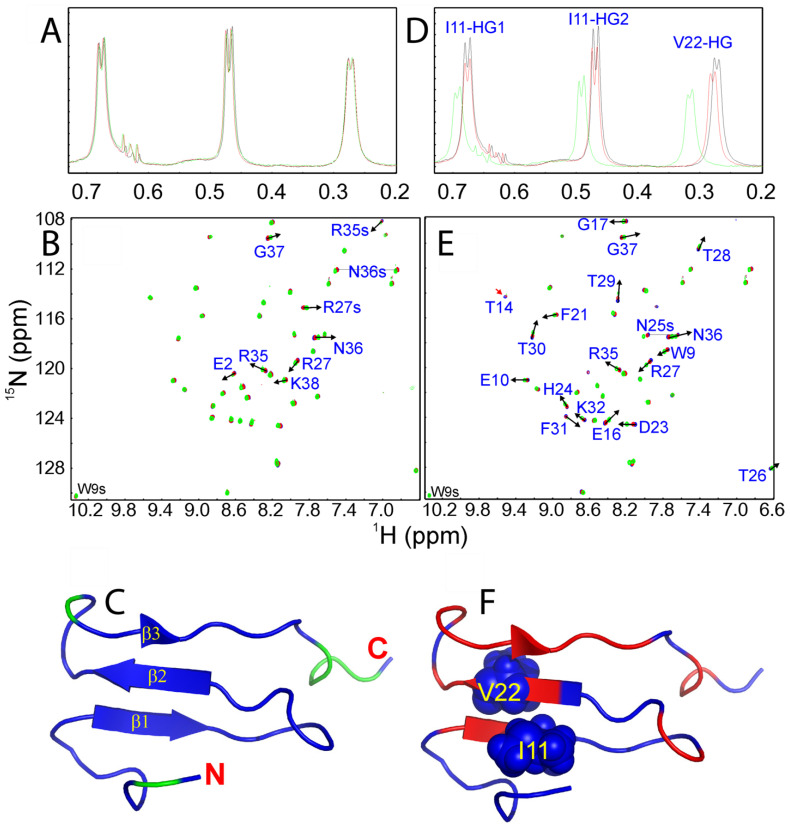
NMR characterization of the binding/perturbation to WW4. One-dimensional ^1^H NMR spectra showing resonance peaks of Ile10 and Val22 methyl groups without (black) and with GdmCl (**A**) or NaSCN (**D**) at 20 mM (red) and 200 mM (green). Two-dimensional ^1^H-^15^N NMR HSQC spectra without (blue) and with GdmCl (**B**) or NaSCN (**E**) at 20 mM (red) and 200 mM (green). (**C**) WW4 structure with residues having significant shifts (CSD >  average  +  STD) of backbone amide protons in the presence of 200 mM GdmCl, colored in green. (**F**) WW4 structure with residues having significant shifts of backbone amide protons in the presence of 200 mM NaSCN, colored in red. Ile10 and Val22 side chains are also displayed in spheres.

**Figure 3:**
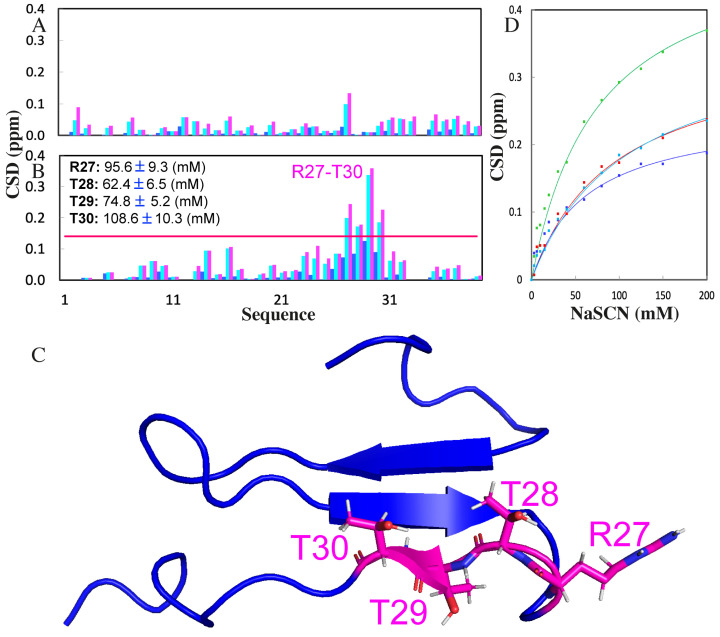
NMR quantification of the perturbations of GdmCl and NaSCN to WW4. (**A**) Chemical shift differences (CSDs) of WW4 in the presence of GdmCl at 20 (blue), 150 (cyan) and 200 (purple) mM. (**B**) Chemical shift differences (CSDs) of WW4 in the presence of NaSCN at 20 (blue), 150 (cyan) and 200 (purple) mM. The red line has a value of 0.14, which is the sum of the average and STD of CSDs in the presence of NaSCN at 200 mM. (**C**) WW4 structure with four residues having CSDs >0.14, displayed in sticks. (**D**) Fitting of residue-specific dissociation constant (Kd) for Arg27 (red), Thr28 (blue), Thr29 (green) and Thr30 (cyan); experimental (dots) and simulated (lines) values for CSDs induced by additions of NaSCN at 3, 6, 10, 15, 20, 30, 40, 60, 80, 100, 125, 150 and 200 mM.

Interestingly, addition of GdmCl even up to 200 mM triggered no significant change in chemical shifts and shapes of Ile11 and Val22 methyl resonances in 1D NMR spectra (Figure 2A), indicating no detectable binding/perturbation of GdmCl with the hydrophobic side chains. Further, HSQC titrations show that GdmCl only weakly interacted with some well-exposed backbone and side chain amide protons (Figure 2B,C and Figure 3A). Briefly, GdmCl even at 200 mM only induced a relatively large shift in the HSQC peaks of Arg27 (Figure 3A), consistent with previous reports that GdmCl has no strong capacity to form hydrogen bonds with most peptide groups [10], but may only weakly pair to residues with positively charged side chains [13,28].

By contrast, the addition of NaSCN led to a significant alteration in both chemical shift and line-broadening of the representative hydrophobic side chain protons (Figure 2D). Interestingly, Ile11 and Val22 are located on the first and second strands, respectively, which constitute the most structured elements of the WW fold (Figure 2F). Moreover, NaSCN also triggers large shifts of many amide protons (Figure 2E and Figure 3B), thus suggesting that NaSCN is able to extensively bind to both hydrophobic and charged groups of WW4. In particular, the HSQC peaks of Arg27, Thr28, Thr29 and Thr30 were significantly shifted (Figure 3B,C). For a folded protein, the salt-induced changes in its NMR signals may result directly from the binding interaction, or/and indirectly from the alterations of conformations and dynamics [22,23,24,25,26,27]. Here, as the shifts of WW4 HSQC peaks occur at millimolar denaturant concentrations, which are far below the concentrations used to globally denature proteins, by assuming that the shifts of HSQC peaks are mostly from the direct binding with denaturant molecules, the dissociation constants (Kd) were fitted out from the CSD data to be 95.6, 62.4, 74.8 and 108.6 mM, respectively, for Arg27, Thr28, Thr29 and Thr30 (Figure 3D). However, small CSD values prevent accurate fitting of the CSD data produced by GdmCl. Furthermore, the HSQC spectral dispersions of WW4 under all five conditions remain almost unchanged, implying that no significant disruption of the tight packing occurs even in the presence of the denaturants up to 200 mM, in a complete agreement with the above near-UV CD results.

### 2.3. Effects of Two Denaturants on Backbone Dynamics of WW4 on the ps-ns Time Scale

NMR spectroscopy offers powerful methods to detect protein dynamics across various time scales. For example, relaxation experiments, including longitudinal relaxation time T1, transverse relaxation time T2 and {^1^H}-^15^N steady-state NOE (hetNOE or hNOE) offer information on dynamics on ps-ns time scales, while Carr–Purcell–Meiboom–Gill (CPMG) relaxation dispersion techniques can probe μs-ms dynamics by measuring how relaxation rates vary with the frequency of applied magnetic fields.

We then decided to assess the dynamic effects of two denaturants on WW4 by acquiring a large set of ^15^N backbone relaxation data for WW4 under the five conditions (Figure S1). As hNOE offers a reliable measure of backbone dynamics on the ps-ns time scale [26,27,29,30,31,32,33,34,35,36,37], the similar hNOE values (Figure S1A) indicate that WW4 has no significant change in ps-ns backbone dynamics under all five conditions. However, differences were observed for T1/T2 values (Figure S1C), implying that WW4 may have distinctive µs-ms dynamics under different conditions.

**Figure 4:**
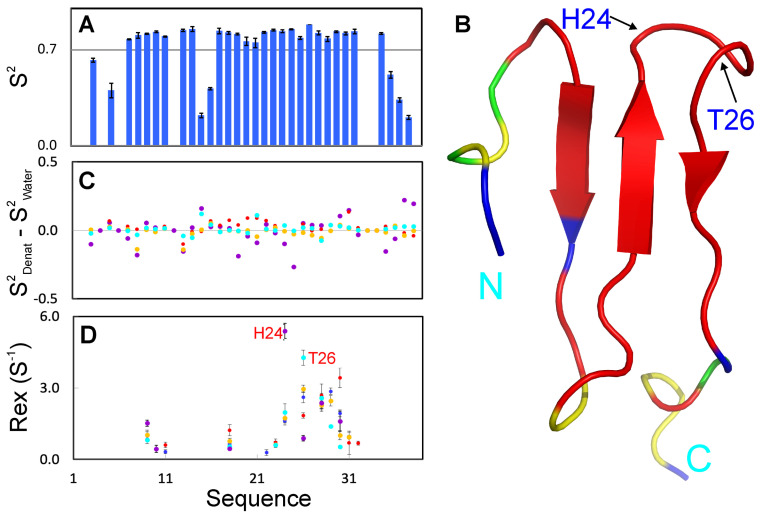
Model-free analysis. (**A**) Squared generalized order parameters (S^2^) of WW4 without denaturant. (**B**) WW4 structure colored based on S^2^ values without denaturant: blue—absence of S^2^ due to the overlap of HSQC peaks or relaxation data of poor quality; green—Proline residues; red—S^2^ > 0.7; and yellow—S^2^ < 0.7. The locations of His24 and Thr26 are also indicated. (**C**) Differences in S^2^ of WW4 with GdmCl at 20 mM (brown) and 200 mM (cyan) or NaSCN at 20 mM (purple) and 200 mM (red) from those without denaturant. (**D**) Residue-specific Rex of WW4 without (blue) and with GdmCl at 20 mM (brown), 200 mM (cyan), or NaSCN at 20 mM (purple) and 200 mM (red).

We then used “Model-free” formalism to analyze the data [26,27,29,30,31,32,33,36,37] with the program Dynamics 3 [32], which includes several extended models in addition to the classic “Model-free approach” [33]. Figure 4A presents the squared generalized order parameters, S^2^, of WW4 without denaturant, which reflect ps-ns conformational dynamics, ranging from zero for high internal motion, to one for completely restricted motion. Except for the terminal residues Asn1-Leu5, Asn36-Ser39 and the loop residues Arg15-Glu16, the other non-Proline residues all have S^2^ > 0.7 (Figure 4A,B), indicating that WW4 is well-folded. On the other hand, the addition of two denaturants only resulted in some slight alterations in the residue-specific S^2^ values (Figure 4C), implying redistribution of ps-ns backbone dynamics. Nevertheless, average S^2^ values are very similar under all five conditions: 0.72 ± 0.01 (no denaturant); 0.71 ± 0.01 (20 mM NaSCN); 0.73 ± 0.01 (200 mM NaSCN); 0.71 ± 0.01 (20 mM GdmCl) and 0.73 ± 0.01 (20 mM GdmCl). The results suggest that the addition of two denaturants even up to 200 mM does not significantly alter the overall backbone dynamics of WW4 on the ps-ns time scale.

### 2.4. Effects of Two Denaturants on Backbone Dynamics of WW4 on the µs-ms Time Scale

Analysis using Dynamics 3 [32] also indicated that some WW4 residues have additional Rex, which reflects conformational exchanges on the µs-ms time scale. As shown in Figure 4D, without denaturant, residues with Rex include Trp9, Ile11, Val18, Val22, Asp23, His24, Thr26, Thr28, Thr29, Thr30 and Phe31. In particular, His24, Thr26, Thr28, Thr29 and Thr30 have Rex > 1 Hz. Markedly, the addition of two denaturants did not significantly change the overall patterns but only altered the Rex value. For example, the addition of GdmCl particularly at 200 mM significantly reduces the Rex values for residues Ile11, Val22, Thr29 and Thr30. By contrast, the addition of NaSCN leads to an increase in the Rex value for residues Trp9, Ile11, Val18, Thr28, Thr30 and Lys32.

**Figure 5:**
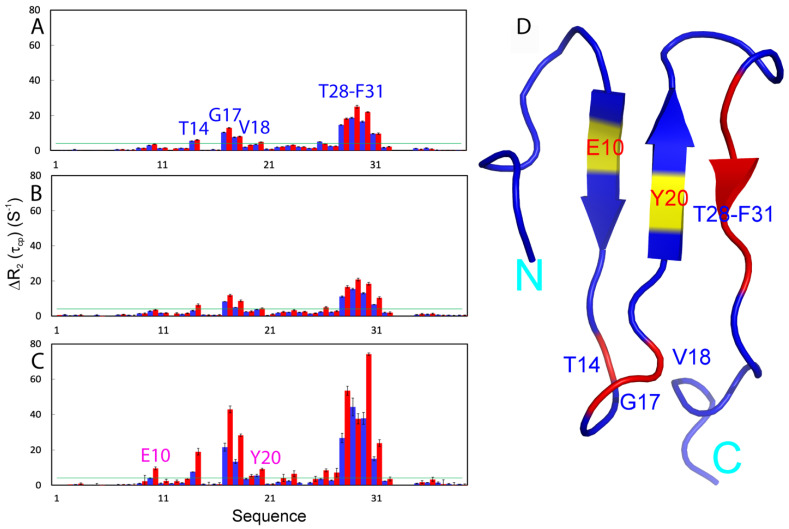
^15^N backbone CPMG relaxation dispersion. Differences in effective transverse relaxation rates (ΔR_2_^eff^) at 80 and 960 Hz, for WW4 without (**A**) and with GdmCl at 200 mM (**B**) or NaSCN at 200 mM (**C**). For WW4 without denaturant, blue bars are for data at 500 MHz while red are for data at 800 MHz. For WW4 with 200 mM GdmCl or NaSCN, blue bars are for data at 500 MHz while red are for data at 800 MHz. (**D**) WW4 structure colored based on ΔR_2_(τ_cp_) without denaturant; red is for residues with ΔR_2_^eff^ > 4 Hz. Glu10 and Tyr20 are specifically colored in yellow as their ΔR_2_^eff^ became >4 Hz upon adding 200 mM NaSCN.

To independently confirm the effects of two denaturants on µs-ms backbone dynamics of WW4, we further performed ^15^N backbone CPMG relaxation dispersion measurements [34,38,39,40,41,42]. As thermal unfolding, NMR binding and relaxation measurements all indicate that the two denaturants only exerted large effects on WW4 at 200 mM, here, we performed CPMG relaxation dispersion measurements on WW4 under three solution conditions, namely in the absence and in the presence of GdmCl or NaSCN at 200 mM at two magnetic fields: 500 MHz and 800 Mz. For WW4 without denaturant, the residues with differences in effective transverse relaxation rates (ΔR_2_^eff^) > 4 Hz include Thr14, Gly17, Val18, Thr28, Thr29, Thr30 and Phe31 (Figure 5A). This pattern is in general consistent with that of Rex derived from “Model-free” analysis. However, His24 and Thr26 with large Rex show no significant CPMG dispersion response. This is commonly observed in previous NMR dynamics studies and many mechanisms might exist to account for the discrepancy between the two measurements [34,38,39,40,41,42]. Here, it is also worthy to note that residues Thr28-Thr30 with large Rex values constitute the most critical binding surface for the Nogo-A peptide [21], implying that the µs-ms conformational exchange might be needed for the functional binding of WW4 via an allosteric mechanism. Structural destabilization in the presence of denaturing agents might activate this allosteric mechanism.

Most unexpectedly, the two denaturants oppositely altered the ΔR_2_^eff^ values of WW4 residues at both 500 MHz and 800 MHz fields. For example, for GdmCl at 200 mM, the ΔR_2_^eff^ values at 800 MHz decreased from 12.9 to 11.8 for Gly17; from 18.1 to 16.6 Hz for Thr28; from 25.0 to 20.8 Hz for Thr29; and from 21.9 to 18.4 Hz for Thr30 (Figure 5B). By contrast, NaSCN at 200 mM dramatically increased the ΔR_2_^eff^ values from 12.9 to 42.8 Hz for Gly17; from 18.1 to 53.5 Hz for Thr28; from 25.0 to 37.6 Hz for Thr29; from 21.9 to 74.2 Hz for Thr30, and from 9.6 to 23.9 Hz for Phe31 (Figure 5C,D). Although NMR relaxation and CPMG relaxation dispersion measurements can be influenced by self-association [43,44], this does not seem to be the case for the present results with WW4, as: (1) it shows no concentration-dependent changes in NMR signals at protein concentrations up to 2 mM [21]. (2) We conducted quick CPMG relaxation dispersion measurements on WW4 in the free state even at 2.0 mM, but found no significant alterations in ΔR_2_^eff^ values. (3) The relatively large changes in R2 values in the presence of denaturants occurred only over the WW4 residues with significant μs-ms dynamics, while the WW4 residues in the well-folded regions but without significant μs-ms dynamics showed no significant increase in R2 values (Figure S1E). (4) In particular, GdmCl and NaSCN, despite both being salts, induce opposite changes in both Rex and ΔR_2_^eff^ values, rendering them incompatible with the possibility that the two denaturants might induce dynamic self-association of WW4 by electrostatic/salt effects. On the other hand, performing CPMG relaxation dispersion measurements on WW4 at a 2.0 mM concentration is impractical due to the proportional need for a corresponding denaturant concentration of 2.0 M, which would lead to the global unfolding because while the mechanism remains incompletely understood, the destabilizing capacity of chemical denaturants not only depends on the protein-to-denaturant ratio but also significantly on the absolute concentration of the denaturants. This phenomenon appears to result at least from the volume-excluding effects and alterations in water/hydration structures imposed by denaturants, which represent key factors in the Hofmeister series [4,8,9,10].

To further gain a quantitative insight, by assuming a two-state conformational exchange, we fitted the data in the absence of denaturant and in the presence of 200 mM GdmCl or NaSCN for the residues with ΔR_2_^eff^ values > 4.0 Hz on both 500 and 800 MHz by the program GUARDD [34,42] to obtain exchange parameters which include the populations of two states, chemical shift differences in the two states and Rex (Table 1). For WW4 without denaturant, the major conformational states have populations ≥ 90% for Thr14, Gly17, Val18 and Thr28-Phe31, which undergo exchanges with a minor state, with Rex ranging from 111.8 to 352.6 Hz. Interestingly, both denaturants significantly increase exchange rate Rex between two states, and in general the increase is more significant for NaSCN than for GdmCl. Noticeably, the ^15^N chemical shift differences between the minor state and native state of these residues are very small, all less than 0.5 ppm, thus implying that the minor state could be highly native-like [43,44,45,46,47,48]. Interestingly, earlier studies unveiled the potential existence of unfolding intermediates preceding the rate-limiting step in protein unfolding, termed the dry molten globular (DMG) intermediate [49,50]. Unlike molten globular (DMG) intermediates, DMG intermediates were proposed to have only slightly altered forms of the native proteins which might be extensively observed during the unfolding of many proteins [51,52,53,54,55]. In this context, the other states of the WW4 domain from the analysis of CPMG dispersion data in the presence of two denaturants appear to be similar to DMG states. However, despite being extremely challenging, comprehensive future characterizations are imperative to confirm this possibility.

## 3. Discussion

The Hofmeister series has been recognized for more than 130 years since the pioneering work of Hofmeister [8]. These effects manifest across various fields including medical, biological, chemical, and industrial sciences. Despite their widespread occurrences, the microscopic mechanisms underpinning the Hofmeister series remains incompletely elucidated. Generally, it is thought that the Hofmeister series emerge from intricate yet specific interplays involving ions and proteins, as well as ions and water molecules in direct contact with proteins—protein hydration. However, due to the extreme complexity of systems where ions, counterions, solvents and cosolutes all have varying roles, delineating the microscopic mechanisms behind the influence of ions on protein stability in the Hofmeister series remains a fundamental challenge [8,9,10,56,57,58,59,60]. In this context, NMR studies on protein conformation, stability, binding and dynamics may offer high-resolution insights into how ions in the Hofmeister series either stabilize or destabilize proteins. Nevertheless, two challenges hinder the visualization of ion effects on most proteins using NMR spectroscopy: (1) Before the global unfolding of most proteins, their hydrophobic side chains are buried and inaccessible to ions at low concentrations. (2) Conversely, when the global unfolding occurs, changes in protein NMR resonances result from a combination of conformational variations and ion–protein interactions, making them nearly indistinguishable.

Recently, we investigated the effects of six salts, including kosmotropic Na_2_SO_4_ and Na_2_HPO_4_, neutral NaCl, as well as chaotropic GdmCl and NaSCN (Figure 1A), on the WW4 domain with a unique fold using CD and NMR spectroscopy. All experiments were conducted at salt concentrations ≤200 mM, where no global unfolding occurred. In this report, we presented the effects of the strongest chaotropic cation, guanidinium, and the strongest chaotropic anion, thiocyanate, on the conformation, thermal stability, binding and backbone dynamics of the WW4 domain across ps-ns and μs-ms time scales. Our findings revealed that, at concentrations ≤200 mM, both denaturants had no discernible effect on the tertiary packing of WW4. However, they did reduce the thermal stability of WW4 by 9 °C and 3.2 °C, respectively. Very unexpectedly, further NMR results have revealed that, even though GdmCl acts as a stronger denaturant, it only minimally interacted with some well-exposed amide protons. In stark contrast, NaSCN, as a weaker denaturant, extensively interacted with both hydrophobic side chain and amide protons. Through NMR relaxation and CPMG relaxation dispersion measurements, known for their high sensitivity in detecting various external effects on proteins [61,62,63,64,65,66,67], we have found that, at concentrations ≤200 mM, GdmCl and NaSCN did not significantly impact the backbone dynamics of WW4 on the ps-ns time scales. However, GdmCl reduced, while NaSCN increased, the Rex and ΔR_2_^eff^ values. Further quantitative analysis of these dynamic data has revealed that the presence of GdmCl and NaSCN at concentrations ≤200 mM altered the conformational exchange of the WW4 domain between the native state and other conformational states, which not only exhibit native-like secondary structures but also native-like tertiary packing.

Our results indicate that prior to the global unfolding, both GdmCl and NaSCN were capable of destabilizing the WW4 domain characterized by distinct interactions and μs-ms dynamics. While it is relatively straightforward to understand that extensive interactions of NaSCN with hydrophobic and hydrophilic groups of WW4 would lead to increased dynamics and reduced thermodynamic stability, it is unexpected to find that GdmCl, as a stronger denaturant, destabilizes the WW4 domain with minimal interactions with the WW4 residues and leads to a reduction in both Rex and ΔR_2_^eff^ values. In the context, our current findings are in complete agreement with the prevailing perspective in the field: when delving into the microscopic effects of Hofmeister salts on proteins, the mechanisms appear to highly depend on the specific contexts of both salts and proteins. On the other hand, at the macroscopic or phenomenological level, these salts exhibit a universal order that applies across diverse fields [8,9,10,58,59]. This raises a fundamental question: what could be the underlying mechanisms responsible for this phenomenon?

Recently, we discovered that, even at a molar ratio of 1:1, ATP can act to antagonize the crowding-induced destabilization of γ-crystallin of the human eye lens [68]. In particular, ATP manifests a general capacity to energy-independently induce folding of ALS-causing C71G-hPFN1 coexisting between the folded and unfolded states at a molar ratio of 1:2, and completely unfolded hSOD1 at 1:8 [27,69]. Remarkably, the inducing ability of ATP comes from its anionic triphosphate, which appears to function by influencing the hydration shell of proteins [27,67,69]. It is well established that the hydration effect is universal and occurs not only with proteins but with all molecules. On the other hand, our comprehension of protein hydration remains poor due to the extreme challenge of experimentally and computationally exploring its structures and dynamics [69,70,71,72,73,74,75,76]. Hence, we propose that the observed effects of GdmCl and NaSCN at very low concentrations may also largely result from their interactions with the hydration shell of the WW4 domain. Indeed, GdmCl has been previously observed to alter the hydration shell of other proteins [74,75].

In summary, our study indicates that even at highly diluted concentrations, GdmCl and NaSCN start to destabilize a protein before the global unfolding occurs, which is associated with distinctive characteristics in interactions and μs-ms backbone dynamics. Quantitative analysis reveals that GdmCl and NaSCN destabilize the WW4 domain by altering the exchanges with minor conformational states. The states display highly native-like secondary structures and tertiary packing but may have some alterations in the structure or dynamics of the hydration shell. Moreover, the relationship between protein thermodynamic stability and dynamics has been a subject of fundamental interest, yet it remains contentious [77,78,79]. Our findings imply that there is no straightforward correlation between the thermodynamic stability of the WW4 domain and its backbone dynamics on both ps-ns and μs-ms time scales. This emphasizes the immense complexity of the interplay between protein dynamics and thermodynamic stability. These results underscore the dawn of the dynamics age in protein science, as AI-based approaches like AlphaFold now enable precise predictions of the structures of proteins and their complexes [80,81].

## 4. Materials and Methods

### 4.1. Expression and Purification of WW4

The expression vector for WW4 was previously constructed [21]. For bacterial expression of the recombinant protein, the vector was transformed into *E. coli* BL21 cells. The cells were then cultured at 37 °C until the OD_600_ value reached 0.6. Subsequently, IPTG was added into the broth to a final concentration of 0.3 mM to induce protein expression for 12 h at 20 °C. Cells were harvested by centrifugation and lysed by sonication in PBS buffer. The recombinant GST-fused WW4 protein was purified by affinity chromatography with glutathione-Sepharose 4B beads (Pharmacia Biotech, Piscataway, NJ, USA) under native conditions. Subsequently, the WW4 domain was released from the GST fusion protein by on-gel thrombin cleavage at room temperature for 3 h, followed by further purification with HPLC on a reverse-phase C18 column (Vydac) eluted with a water–acetonitrile system by gradually increasing the acetonitrile concentration. To isotope-label the WW4 protein for ^1^H-^15^N NMR HSQC experiments, the recombinant protein was prepared with a similar protocol except the cells were grown in M9 medium with addition of (^15^NH4)_2_SO_4_ for ^15^N labeling [21].

### 4.2. Circular Dichroism (CD) Experiments

Non-specific noise was extremely high over the far-UV region in the presence of GdmCl and NaSCN. Therefore, in the present study, we characterized the conformation and thermal stability of WW4 by monitoring the near-UV region (260–360 nm). All near-UV CD spectra were collected in 1 mM Tris-HCl at pH 6.4 in the absence and in the presence of two denaturants at different concentrations, on a Jasco J-810 spectropolarimeter equipped with a thermal controller as described previously [20,23] with a protein concentration of 250 µM at 25 °C, using a 1 mm path length cuvette with a 0.1 nm spectral resolution. Data from five independent scans were added and averaged. Thermal unfolding was performed on the same samples used for obtaining the above near-UV CD spectra with temperatures ranging from 20 to 90 °C [26].

### 4.3. NMR Titration of GdmCl and NaSCN to WW4

One WW4 stock sample was prepared by dissolving the protein powder in 1 mM Tris-HCl buffer to a final concentration of 250 µM and its pH was adjusted to 6.4 by adding either diluted sodium hydroxide or hydrogen chloride, and it was subsequently split into individual NMR samples for titrations. The pH values of two denaturant solutions (1 M) in 1 mM Tris-HCl buffer were also adjusted to 6.4.

All NMR titration experiments were collected at 25 °C on an 800 MHz Bruker Avance spectrometer equipped with a shielded cryoprobe as described previously [21,24,25,26,27]. During titrations, series of one-dimensional ^1^H and two-dimensional ^1^H-^15^N HSQC spectra were acquired on the ^15^N-labeled WW4 domain at a concentration of 250 µM in the absence or in the presence of GdmCl and NaSCN at varying salt concentrations (3, 6, 10, 20, 30, 40, 60, 80, 100, 125, 150 and 200 mM) as we previously conducted with 14 salts [24]. The pH values of the NMR samples for each titration were measured before and after titrations with 200 mM salts and no detectable difference was found. NMR data were processed with NMRPipe [82] and subsequently analyzed with NMRView [83].

### 4.4. Calculation of CSD and Data Fitting to Obtain Kd

To calculate chemical shift difference (CSD), HSQC spectra were superimposed for the ^15^N-labeled WW4 domain collected in the free state and in the presence of GdmCl and NaSCN at different concentrations. Subsequently, the shifted HSQC peaks could be identified and further assigned to the corresponding WW4 residues based on the sequential assignment we previously obtained [21]. The CSD was calculated by an integrated index with the following formula [84,85]:

CSD = ((Δ^1^H)^2^ + (Δ^15^N/4)^2^)^1/2^ (1)

In order to obtain the residue-specific dissociation constant (Kd), we fitted the shift traces of the residues with large shifts of HSQC peaks (CSD > average + STD) by using the one-binding-site model with the following formula [23,24,25]:

CSD_obs_ = CSD_max_{([P] + [L] + Kd) − [[P] + [L] + (Kd)^2^ − [P][L]]^1/2^}/2[P] (2)

Here, [P] and [L] are molar concentrations of WW4 and GdmCl and NaSCN, respectively.

### 4.5. NMR Characterization of ^15^N Backbone Dynamics on the ps-ns Time Scale

^15^N backbone T1 and T1ρ relaxation times and {^1^H}-^15^N steady state NOE intensities were collected for WW4 with a concentration of 250 µM at pH 6.4 under five conditions, namely WW4 without denaturant, with 20 mM GdmCl or NaSCN as well as with 200 mM GdmCl or NaSCN, on a Bruker DRX 500 MHz spectrometer equipped with pulse field gradient units at 25 °C [26,27,86,87]. Relaxation time T1 was determined by collecting HSQC spectra with delays of 10, 80, 200, 320, 360, 420 and 500 ms using a recycle delay of 1 s, with a repeat at 200 ms. Relaxation time T1ρ was measured by collecting spectra with delays of 1, 30, 60, 90, 110, 130, 150 and 180 ms using a spin-lock power of 1.6 kHz and a 2.5 s recycle delay with a repeat at 90 ms. {^1^H}-^15^N steady-state NOEs were obtained by recording spectra with and without ^1^H presaturation, a duration of 3 s and a relaxation delay of 6 s.

NMR relaxation data were analyzed by “Model-free” formalism with protein dynamics software suites [36,37] Briefly, relaxation of protonated heteronuclei is dominated by the dipolar interaction with the directly attached ^1^H spin and by the chemical shift anisotropy mechanism [29,30,36,37]. Relaxation parameters are given by:

(3a) R1=d24JωH−ωX+3JωX+6JωH+ωX+c2J(ωX)

(3b) R2=d284J0+JωH−ωX+3JωX+6JωH+6JωH+ωX+(c26)4J0+3JωX+Rex

(3c) $$NOE=1+\left(d^{2}/4R_{1}\right)\left(\gamma_{X}/\gamma_{H}\right)[6J\left(\omega_{H}+\omega_{X}\right)-J(\omega_{H}-\omega_{X})]$$

Here, $d=\mu_{0}h\gamma_{X}\gamma_{H}<r_{XH}^{-3}>/8\pi^{2}$, $c=\omega_{X}∆\sigma /\sqrt{3}$, $\mu_{0}$ is the permeability of free space; *h* is Planck’s constant; $\gamma_{X}{\;\gamma }_{H}$ are the gyromagnetic ratios of ^1^H and the X spin (X = ^13^C or ^15^N), respectively; $\gamma_{XH}$ is the X-H bond length; $\omega_{H}$ and $\omega_{X}$ are the Larmor frequencies of ^1^H and X spins, respectively; and Δσ is the chemical shift anisotropy of the X spin.

Model-free formalism, as previously established and further extended [29,30,32,33,34,35,36], determines the amplitudes and time scales of the intramolecular motions by modeling the spectral density function, *J*(*ω*), as

(4) $$J\left(\omega \right)=\frac{2}{5}\left[\frac{S^{2}\tau_{m}}{1+{\left(\omega \tau_{m}\right)}^{2}}+\frac{\left({S_{f}}^{2}-S^{2}\right)\tau }{1+{\left(\omega \tau \right)}^{2}}\right]\;\;\;\;\;\;\;\;\;\;\;\;\;\;=\frac{2}{5}{S_{f}}^{2}[\frac{{S_{s}}^{2}\tau_{m}}{1+{\left(\omega \tau_{m}\right)}^{2}}+\frac{(1-{S_{s}}^{2})\tau }{1+{\left(\omega \tau \right)}^{2}}]$$

Here, $\tau =\tau_{s}\tau_{m}/\left(\tau_{s}+\tau_{m}\right)$, $\tau_{m}$ is the isotropic rotational correlation time of the molecule, $\tau_{s}$ is the effective correlation time for internal motions, $S^{2}={S_{f}}^{2}{S_{s}}^{2}$ is the square of the generalized order parameter characterizing the amplitude of the internal motions, and ${S_{f}}^{2}$ and ${S_{s}}^{2}$ are the squares of the order parameters for the internal motions on the fast and slow time scales, respectively.

In order to allow for diverse protein dynamics, several forms of the spectral density function, based on various models of the local motion, were utilized, which include the original Lipari–Szabo approach, assuming fast local motion characterized by the parameters *S*^2^ and *τ_loc_*, extended model-free treatment, including both fast (${S_{fast}}^{2},\;\tau_{fast}$) and slow (${S_{slow}}^{2},\;\tau_{slow}$) reorientations for the NH bond ($\tau_{fast}\ll \tau_{slow}<\tau_{c}$), and could also allow for slow, micro- to milli-second dynamics resulting in a conformational exchange contribution, *R_ex_*.

In the present study, the WW4 NMR structure (2OP7) with the lowest energy was used for “Model-free” analysis. For the HSQC spectra of WW4 under five conditions, all peaks are well-separated and thus data are of high quality, except for the overlap of the Arg12 and Asp33 peaks (Figure 2). Here, the overall rotational diffusion tensors and τ_c_ of WW4 under five conditions were determined by ROTDIF [36] while “Model-free” analysis of relaxation data was performed by the software DYNAMICS, which includes the classic and extended “Model-free” models [32]. τ_c_, equivalent to 1/(6D_iso_) in nanoseconds, represents the overall rotational correlation time, which is calculated directly from the relaxation data. We analyzed the relaxation data with three overall models, namely isotropic, axially symmetric and fully anisotropic models and subsequently the axially symmetric model was found to best describe WW4 under different conditions.

### 4.6. NMR Characterization of ^15^N Backbone Dynamics on the µs-ms Time Scale

^15^N transverse relaxation dispersion experiments for WW4 with a concentration of 250 µM under three conditions were acquired on DRX 500 and Bruker Avance 800 MHz spectrometers equipped with a *z*-axis gradient cryoprobe at 25 °C. A constant time delay (*T_CP_* = 50 ms) was used with a series of CPMG frequencies, ranging from 40, 80, 120, 160, 200, 240, 280, 320, 400, 480, 560, 640, 720, 800 to 960 Hz, with a repeat at 120 Hz. A reference spectrum without the CPMG block was acquired to calculate the effective transverse relaxation rate by the following equation:

(5) $$R_{2}^{eff}=-\ln(I(\nu_{CPMG})/I_{0})/T_{CP}$$

where *I*(*ν_CPMG_*) is the peak intensity on the difference CPMG frequency and *I*_0_ is the peak intensity in the reference spectra.

The two-field (500 and 800 MHz) data for WW4 in the absence and in the presence of 200 mM GdmCl and NaSCN were analyzed by assuming a two-state conformational exchange, using the program GUARDD with the equation [44]:

(6a) $$R_{2}^{Eff}\left(\;\frac{1}{2\delta }\;\right)=R\left(\lambda_{1}\right)-\frac{ln(Q)}{4n\delta }$$

Here:

(6b) $$\lambda_{1}=R_{2}^{0}+\frac{1}{2}\;\left(k_{ex}-\left(\frac{1}{2\delta }\right)\;{cosh}^{-1}\left(D_{+}\cosh\left(\eta_{+}\right)-\;D_{-}\cosh\left(\eta_{-}\right)\right)\right)\;$$

(6c) $$D_{\pm }=\frac{1}{2}\;\;\left(\frac{\Psi +2{\Delta \omega }_{x}^{2}}{{\;(\Psi^{2}+\zeta^{2})}^{1/2}}\;\;\pm \;1\right)\;\;\;\;$$

(6d) $$\eta_{\pm }=\sqrt{2}\;\delta {\left({\;\left(\Psi^{2}+\zeta^{2}\right)}^{\frac{1}{2}}\pm \Psi \right)}^{1/2}$$

(6e) $$\Psi ={\left(i\Delta \omega_{H}+(P_{A}-P_{B})k_{ex}\right)}^{2}-{\Delta \omega }_{C}^{2}+4P_{A}P_{B}K_{ex}^{2}$$

(6f) $$\zeta =-2\Delta \omega_{C}(i\Delta \omega_{H}+\left(P_{A}-P_{B}\right)k_{ex})$$

(6g) $$Q=R\left(\;1-m_{D}^{\;2}+m_{D}m_{Z}-m_{Z}^{\;2\;}+\frac{1}{2}\left(m_{D}{+m}_{Z}\right){\left(\frac{P_{B}}{P_{A}}\right)}^{1/2}\right)$$

(6h) $$m_{D}=\frac{{ik}_{ex}{\left(P_{B}P_{A}\right)}^{1/2}}{d_{+}z_{+}}\;\left(z_{+}+2\;{\Delta \omega }_{X}\;\left(\frac{sin(z_{+}\delta )\;}{sin({{\;(d}_{+}+z}_{+})\delta )}\right)\;\right)$$

(6i) $$m_{z}=\frac{{ik}_{ex}{\left(P_{B}P_{A}\right)}^{1/2}}{d_{-}z_{-}}\;\left(d_{-}-2\;{\Delta \omega }_{X}\;\left(\frac{sin(d_{-}\delta )\;}{sin({{\;(d}_{-}+z}_{-})\delta )}\right)\;\right)$$

(6j) $$d_{\pm }=\left(\Delta \omega_{H}+\Delta \omega_{X}\right)\pm {ik}_{ex}$$

(6k) $$z_{\pm }=\left(\Delta \omega_{H}-\Delta \omega_{X}\right)\pm {ik}_{ex}$$

The above fitting equations are for MQ dispersions, which can simplify to SQ dispersions provided Δω_H_ = 0 [44]. The obtained parameters defining conformational exchanges are presented in Table 1.

## Figures and Tables

**Table 1:** Parameters defining the conformational exchanges.

		WW4			GdmCl			NaSCN	
Residues	Δω ^a^ (ppm)	Pa ^b^ (%)	Kex ^c^ (Hz)	Δω (ppm)	Pa (%)	Kex (Hz)	Δω (ppm)	Pa (%)	Kex (Hz)
E10							0.2 ± 0.08	1.9 ± 0.1	1287.0 ± 74.0
T14	0.25 ± 0.02	1.6 ± 0.1	352.6 ± 149.1	0.19 ± 0.03	1.4 ± 0.1	732.0 ± 156.0	0.16 ± 0.03	5.4 ± 0.4	904.7 ± 177.9
G17	0.33 ± 0.02	9.4 ± 0.9	111.8 ± 107.8	0.25 ± 0.01	2.4 ± 0.6	612.4 ± 80.9	0.25 ± 0.01	7.6 ± 0.6	918.1 ± 50.1
V18	0.28 ± 0.02	2.5 ± 0.6	308.9 ± 129.2	0.21 ± 0.02	1.8 ± 0.3	724.8 ± 114.1	0.19 ± 0.01	6.2 ± 0.2	923.0 ± 85.6
Y20							0.22 ± 0.01	1.9 ± 0.3	632 ± 81.4
T28	0.35 ± 0.02	7.5 ± 0.7	199.4 ± 130.3	0.27 ± 0.15	2.9 ± 0.4	819.6 ± 87.9	0.23 ± 0.01	10.1 ± 0.9	1064.2 ± 78.0
T29	0.44 ± 0.03	8.3 ± 1.0	254.4 ± 169.6	0.35 ± 0.02	3.1 ± 0.4	878.7 ± 106.7	0.34 ± 0.04	12.7 ± 1.1	422.2 ± 216.1
T30	0.33 ± 0.02	10.0 ± 1.2	166.1 ± 118.6	0.27 ± 0.02	3.4 ± 0.4	712.2 ± 85.1	0.25 ± 0.01	11.1 ± 0.9	967.2 ± 83.2
F31	0.25 ± 0.02	3.5 ± 0.7	269.3 ± 110.8	0.21 ± 0.01	2.2 ± 0.3	665.4 ± 79.2	0.24 ± 0.02	4.9 ± 0.4	571.1 ± 105.0

^a^ Chemical shift difference between the exchanging A state and B states (ppm). ^b^ Population of the minor state. ^c^ Total exchange rate between A and B states.

## Data Availability

The data presented in this study are available in the manuscript and Supplementary Materials.

## Supplementary Materials

The following supporting information can be downloaded at: https://www.mdpi.com/article/10.3390/ijms25137344/s1.

## References

[1] Anfinsen C.B. (1973). Principles that govern the folding of protein chains. Science.

[2] Baldwin R.L. (2005). Early days of studying the mechanism of protein folding. Protein Folding Handbook.

[3] Kim D.E., Gu H., Baker D. (1998). The sequences of small proteins are not extensively optimized for rapid folding by natural selection. Proc. Natl. Acad. Sci. USA.

[4] Baldwin R.L. (1996). How Hofmeister ion interactions affect protein stability. Biophys. J..

[5] Palmer A.G. (2001). NMR probes of molecular dynamics: Overview and comparison with other techniques. Annu. Rev. Biophys. Biomol. Struct..

[6] Sekhar A., Kay L.E. (2019). An NMR View of Protein Dynamics in Health and Disease. Annu. Rev. Biophys..

[7] Gonzalez N.A., Li B.A., McCully M.E. (2022). The stability and dynamics of computationally designed proteins. Protein Eng. Des. Sel..

[8] Hofmeister F. (1888). Zur Lehre von der Wirkung der Salze. Arch. Exp. Pathol. Pharmakol..

[9] Salis B.W. (2014). Ninham Models and mechanisms of Hofmeister effects in electrolyte solutions, and colloid and protein systems revisited. Chem. Soc. Rev..

[10] Okur H.I., Hladílková J., Rembert K.B., Cho Y., Heyda J., Dzubiella J., Cremer P.S., Jungwirth P. (2017). Beyond the Hofmeister Series: Ion-Specific Effects on Proteins and Their Biological Functions. J. Phys. Chem. B.

[11] Lim W.K., Rosgen J., Englander S.W. (2009). Urea, but not guanidinium, destabilizes proteins by forming hydrogen bonds to the peptide group. Proc. Natl. Acad. Sci. USA.

[12] Street T.O., Bolen D.W., Rose G.D. (2006). A molecular mechanism for osmolyte-induced protein stability. Proc. Natl. Acad. Sci. USA.

[13] O’Brien E.P., Dima R.I., Brooks B., Thirumalai D. (2007). Interactions between hydrophobic and ionic solutes in aqueous guanidinium chloride and urea solutions: Lessons for protein denaturation mechanism. J. Am. Chem. Soc..

[14] Kubíčková A., Křížek T., Coufal P., Wernersson E., Heyda J., Jungwirth P. (2011). Guanidinium cations pair with positively charged arginine side chains in water. J. Phys. Chem. Lett..

[15] Bennion B.J., Daggett V. (2003). The molecular basis for the chemical denaturation of proteins by urea. Proc. Natl. Acad. Sci. USA.

[16] Mason P.E., Neilson G.W., Dempsey C.E., Barnes A.C., Cruickshank J.M. (2003). The hydration structure of guanidinium and thiocyanate ions: Implications for protein stability in aqueous solution. Proc. Natl. Acad. Sci. USA.

[17] Robinson D.R., Jencks W.P. (1965). The effect of compounds of the urea-guanidinium class on the activity coefficient of acetyltetraglycine ethyl ester and related compounds. J. Am. Chem. Soc..

[18] England J.L., Haran G. (2011). Role of solvation effects in protein denaturation: From thermodynamics to single molecules and back. Annu. Rev. Phys. Chem..

[19] TiradoRives J., Orozco M., Jorgensen W.L. (1997). Molecular dynamics simulations of the unfolding of barnase in water and 8 m aqueous urea. Biochemistry.

[20] Caflisch A., Karplus M. (1999). Structural details of urea binding to barnase: A molecular dynamics analysis. Structure.

[21] Qin H., Pu H.X., Li M., Ahmed S., Song J. (2008). Identification and structural mechanism for a novel interaction between a ubiquitin ligase WWP1 and Nogo-A, a key inhibitor for central nervous system regeneration. Biochemistry.

[22] Zuiderweg E.R. (2002). Mapping protein–protein interactions in solution by NMR spectroscopy. Biochemistry.

[23] Williamson M.P. (2013). Using chemical shift perturbation to characterise ligand binding. Prog. Nucl. Magn. Reson. Spectrosc..

[24] Miao L., Qin H., Koehl P., Song J. (2011). Selective and specific ion binding on proteins at physiologically-relevant concentrations. FEBS Lett..

[25] Kang J., Lim L., Song J. (2019). ATP binds and inhibits the neurodegeneration-associated fibrillization of the FUS RRM domain. Commun. Biol..

[26] Lu Y., Lim L., Song J. (2017). RRM domain of ALS/FTD-causing FUS characteristic of irreversible unfolding spontaneously self-assembles into amyloid fibrils. Sci. Rep..

[27] Kang J., Lim L., Song J. (2023). ATP induces folding of ALS-causing C71G-hPFN1 and nascent hSOD1. Commun. Chem..

[28] Zarrine-Afsar A., Mittermaier A., Kay L.E., Davidson A.R. (2006). Protein stabilization by specific binding of guanidinium to a functional arginine-binding surface on an SH3 domain. Protein Sci..

[29] Farrow N.A., Muhandiram R., Singer A.U., Pascal S.M., Kay C.M., Gish G., Shoelson S.E., Pawson T., Forman-Kay J.D., Kay L.E. (1994). Backbone dynamics of a free and phosphopeptide-complexed Src homology 2 domain studied by 15N NMR relaxation. Biochemistry.

[30] Clore G.M., Driscoll P.C., Wingfield P.T., Gronenborn A.M. (1990). Analysis of the backbone dynamics of interleukin-1 b using two-dimensional inverse detected heteronuclear 15N–1H NMR spectroscopy. Biochemistry.

[31] Dyson H.J., Wright P.E. (2004). Unfolded proteins and protein folding studied by NMR. Chem. Rev..

[32] Fushman D., Cahill S., Cowburn D. (1997). The main-chain dynamics of the dynamin pleckstrin homology (PH) domain in solution: Analysis of 15N relaxation with monomer/dimer equilibration. J. Mol. Biol..

[33] Lipari G., Szabo A. (1982). Model-free approach to the interpretation of Nuclear Magnetic Resonance relaxation in macromolecules. 1. Theory and range of validity. J. Am. Chem. Soc..

[34] Qin H., Lim L., Song J. (2015). Dynamic principle for designing antagonistic/agonistic molecules for EphA4 receptor, the only known ALS modifier. ACS Chem. Biol..

[35] Qin H., Lim L.Z., Wei Y., Song J. (2014). TDP-43 N terminus encodes a novel ubiquitin-like fold and its unfolded form in equilibrium that can be shifted by binding to ssDNA. Proc. Natl. Acad. Sci. USA.

[36] Walker O., Varadan R., Fushman D. (2004). Efficient and accurate determination of the overall rotational diffusion tensor of a molecule from 15N relaxation data using computer program ROTDIF. J. Magn. Reson..

[37] Hall J.B., Fushman D. (2003). Characterization of the overall and local dynamics of a protein with intermediate rotational anisotropy: Differentiating between conformational exchange and anisotropic diffusion in the B3 domain of protein G. J. Biomol. NMR.

[38] Mulder F.A., Hon B., Mittermaier A., Dahlquist F.W., Kay L.E. (2002). Slow internal dynamics in proteins: Application of NMR relaxation dispersion spectroscopy to methyl groups in a cavity mutant of T4 lysozyme. J. Am. Chem. Soc..

[39] Palmer A.G. (2004). NMR characterization of the dynamics of biomacromolecules. Chem. Rev..

[40] Kleckner I.R., Foster M.P. (2011). An introduction to NMR-based approaches for measuring protein dynamics. Biochim. Biophys. Acta.

[41] Millet O., Loria J.P., Kroenke C.D., Pons M., Palmer A.G. (2000). The static magnetic field dependence of chemical exchange line broadening defines the NMR chemical shift time scale. J. Am. Chem. Soc..

[42] Kleckner I.R., Foster M.P. (2012). GUARDD: User-friendly MATLAB software for rigorous analysis of CPMG RD NMR data. J. Biomol. NMR.

[43] Pfuhl M., Chen H.A., Kristensen S.M., Driscoll P.C. (1999). NMR exchange broadening arising from specific low affinity protein self-association: Analysis of nitrogen-15 nuclear relaxation for rat CD2 domain 1. J. Biomol. NMR.

[44] Akerud T., Thulin E., Van Etten R.L., Akke M. (2002). Intramolecular dynamics of low molecular weight protein tyrosine phosphatase in monomer-dimer equilibrium studied by NMR: A model for changes in dynamics upon target binding. J. Mol. Biol..

[45] Balbach J., Forge V., Lau W.S., van Nuland N.A., Brew K., Dobson C.M. (1996). Protein folding monitored at individual residues during a two-dimensional NMR experiment. Science.

[46] Korzhnev D.M., Salvatella X., Vendruscolo M., Di Nardo A.A., Davidson A.R., Dobson C.M., Kay L.E. (2004). Low-populated folding intermediates of Fyn SH3 characterized by relaxation dispersion NMR. Nature.

[47] Schulman B.A., Kim P.S., Dobson C.M., Redfield C. (1997). A residue-specific NMR view of the non-cooperative unfolding of a molten globule. Nat. Struct. Biol..

[48] Song J., Bai P., Luo L., Peng Z.Y. (1998). Contribution of individual residues to formation of the native-like tertiary topology in the alpha-lactalbumin molten globule. J. Mol. Biol..

[49] Kiefhaber T., Labhardt A.M., Baldwin R.L. (1995). Direct NMR evidence for an intermediate preceding the rate-limiting step in the unfolding of ribonuclease A. Nature.

[50] Jha S.K., Marqusee S. (2014). Kinetic evidence for a two-stage mechanism of protein denaturation by guanidinium chloride. Proc. Natl. Acad. Sci. USA.

[51] Song J., Jamin N., Gilquin B., Vita C., Ménez A. (1999). A gradual disruption of tight side-chain packing: 2D 1H-NMR characterization of acid-induced unfolding of CHABII. Nat. Struct. Biol..

[52] Wei Z., Song J. (2005). Molecular mechanism underlying the thermal stability and pH-induced unfolding of CHABII. J. Mol. Biol..

[53] Bhatia S., Udgaonkar J.B. (2022). Heterogeneity in protein folding and unfolding reactions. Chem. Rev..

[54] Holehouse A.S., Pappu R.V. (2018). Collapse Transitions of Proteins and the Interplay Among Backbone, Sidechain, and Solvent Interactions. Annu. Rev. Biophys..

[55] Mishra P., Jha S.K. (2022). The native state conformational heterogeneity in the energy landscape of protein folding. Biophys. Chem..

[56] Zhang Y., Cremer P.S. (2006). Interactions between macromolecules and ions: The Hofmeister series. Curr. Opin. Chem. Biol..

[57] Mráček A., Varhaníková J., Lehocký M., Gřundělová L., Pokopcová A., Velebný V. (2008). The influence of Hofmeister series ions on hyaluronan swelling and viscosity. Molecules.

[58] Gregory K.P., Wanless E.J., Webber G.B., Craig V.S.J., Page A.J. (2021). The electrostatic origins of specific ion effects: Quantifying the Hofmeister series for anions. Chem. Sci..

[59] Gregory K.P., Elliott G.R., Robertson H., Kumar A., Wanless E.J., Webber G.B., Craig V.S.J., Andersson G.G., Page A.J. (2022). Understanding specific ion effects and the Hofmeister series. Phys. Chem. Chem. Phys..

[60] Kukic P., O’Meara F., Hewage C., Nielsen J.E. (2013). Coupled effect of salt and pH on proteins probed with NMR spectroscopy. Chem. Phys. Lett..

[61] Ding K., Louis J.M., Gronenborn A.M. (2004). Insights into conformation and dynamics of protein GB1 during folding and unfolding by NMR. J. Mol. Biol..

[62] Jarymowycz V.A., Stone M.J. (2006). Fast time scale dynamics of protein backbones: NMR relaxation methods, applications, and functional consequences. Chem. Rev..

[63] Loria J.P., Berlow R.B., Watt E.D. (2008). Characterization of enzyme motions by solution NMR relaxation dispersion. Acc. Chem. Res..

[64] Palmer A.G. (2009). A topical issue: NMR investigations of molecular dynamics. J. Biomol. NMR.

[65] Boehr D.D., McElheny D., Dyson H.J., Wright P.E. (2010). Millisecond timescale fluctuations in dihydrofolate reductase are exquisitely sensitive to the bound ligands. Proc. Natl. Acad. Sci. USA.

[66] Baldwin A.J., Kay L.E. (2009). NMR spectroscopy brings invisible protein states into focus. Nat. Chem. Biol..

[67] Nucci N.V., Pometun M.S., Wand A.J. (2011). Mapping the hydration dynamics of ubiquitin. J. Am. Chem. Soc..

[68] He Y., Kang J., Song J. (2020). ATP antagonizes the crowding-induced destabilization of the human eye-lens protein γS-crystallin. Biochem. Biophys. Res. Commun..

[69] Song J. (2021). Adenosine triphosphate energy-independently controls protein homeostasis with unique structure and diverse mechanisms. Protein Sci..

[70] Baldwin R.L. (2014). Dynamic hydration shell restores Kauzmann’s 1959 explanation of how the hydrophobic factor drives protein folding. Proc. Natl. Acad. Sci. USA.

[71] Levy Y., Onuchic J.N. (2006). Water mediation in protein folding and molecular recognition. Annu. Rev. Biophys. Biomol. Struct..

[72] Song J. (2009). Insight into “insoluble proteins” with pure water. FEBS Letts..

[73] Laage D., Elsaesser T., Hynes J.T. (2017). Water dynamics in the hydration shells of biomolecules. Chem. Rev..

[74] Mazza M.G., Stokely K., Pagnotta S.E., Bruni F., Stanley H.E., Franzese G. (2011). More than one dynamic crossover in protein hydration water. Proc. Natl. Acad. Sci. USA.

[75] Scott J.N., Nucci N.V., Vanderkooi J.M. (2008). Changes in water structure induced by the guanidinium cation and implications for protein denaturation. J. Phys. Chem. A.

[76] Roche J., Caro J.A., Norberto D.R., Barthe P., Roumestand C., Schlessman J.L., Garcia A.E., García-Moreno E.B., Royer C.A. (2012). Cavities determine the pressure unfolding of proteins. Proc. Natl. Acad. Sci. USA.

[77] Spyracopoulos L. (2005). Thermodynamic interpretation of protein dynamics from NMR relaxation measurements. Protein Pept. Lett..

[78] Doan-Nguyen V., Loria J.P. (2007). The effects of cosolutes on protein dynamics: The reversal of denaturant-induced protein fluctuations by trimethylamine N-oxide. Protein Sci..

[79] Kamerzell T.J., Middaugh C.R. (2008). The complex inter-relationships between protein flexibility and stability. J. Pharm. Sci..

[80] Jumper J., Evans R., Pritzel A., Green T., Figurnov M., Ronneberger O., Tunyasuvunakool K., Bates R., Žídek A., Potapenko A. (2021). Highly accurate protein structure prediction with AlphaFold. Nature.

[81] Abramson J., Adler J., Dunger J., Evans R., Green T., Pritzel A., Ronneberger O., Willmore L., Ballard A.J., Bambrick J. (2023). Accurate structure prediction of biomolecular interactions with AlphaFold 3. Nature.

[82] Delaglio F., Grzesiek S., Vuister G.W., Zhu G., Pfeifer J., Bax A. (1995). NMRPipe: A multidimensional spectral processing system based on UNIX pipes. J. Biomol. NMR.

[83] Johnson B.A., Blevins R.A. (1994). NMRView: A computer program for the visualization and analysis of NMR data. J. Biomol. NMR.

[84] Ortega-Roldan J.L., Blackledge M., Jensen M.R. (2018). Characterizing Protein-Protein Interactions Using Solution NMR Spectroscopy. Methods Mol. Biol..

[85] Giri M., Gupta P., Maulik A., Gracias M., Singh M. (2022). Structure and DNA binding analysis of AT-rich interaction domain present in human BAF-B specific subunit BAF250b. Protein Sci..

[86] Fan D., Zheng Y., Yang D., Wang J. (2003). NMR solution structure and dynamics of an exchangeable apolipoprotein, Locusta migratoria apolipophorin III. J. Biol. Chem..

[87] Ran X., Qin H., Liu J., Fan J.S., Shi J., Song J. (2008). NMR structure and dynamics of human ephrin-B2 ectodomain: The functionally critical C-D and G-H loops are highly dynamic in solution. Proteins.

