# Supplementary material for: NMR Dynamic View of the Destabilization of WW4 Domain by Chaotropic GdmCl and NaSCN
Source: Int J Mol Sci. 2024 Jul 4;25(13):7344. doi: 10.3390/ijms25137344 (PMC11242413; doi:10.3390/ijms25137344)
Supplement: Supplementary file 1 [file ijms-25-07344-s001.zip › ijms-3070057-supplementary.pdf]

Supplementary Figure

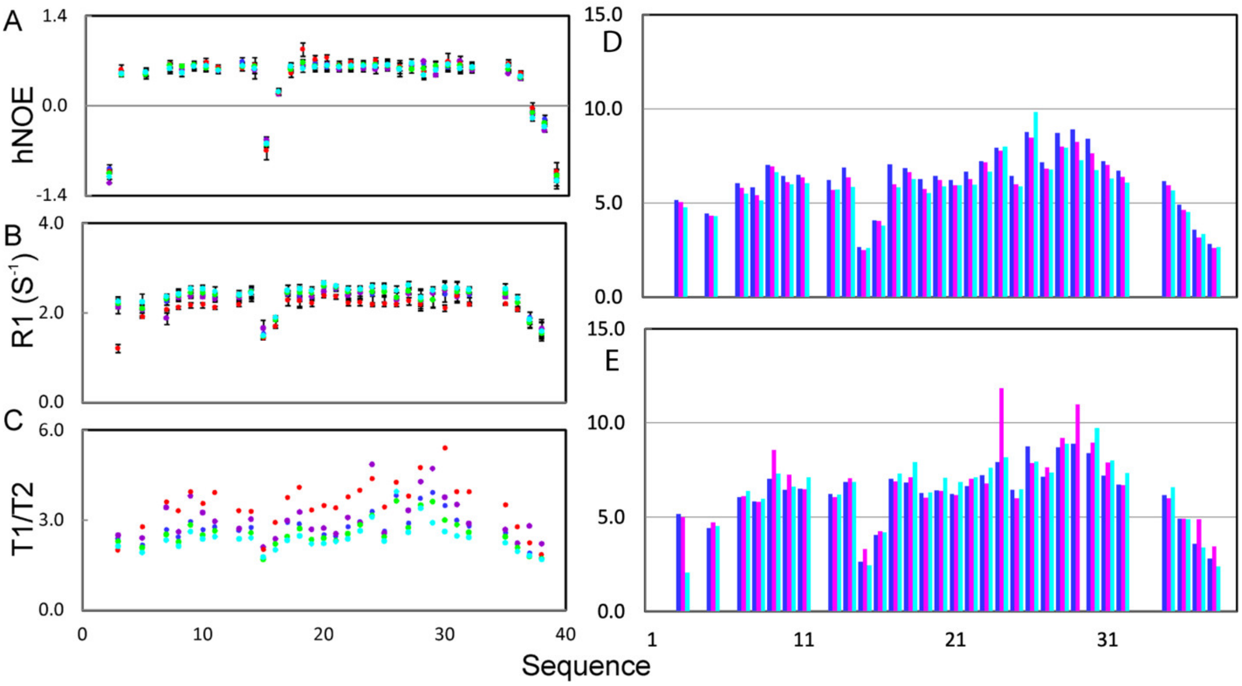

Figure S1. ^{15}N backbone relaxation data of WW4 under five conditions.
{^{1}H}-^{15}N steady state NOE intensities (hNOE). (B) Inverse of longitudinal relaxation time, T1 (R1). (C) T1 divided by T2 (transverse relaxation time). Blue: WW4 without denaturant; green: with GdmCl at 20 mM; cyan: with GdmCl at 200 mM; purple: with NaSCN at 20 mM; red: with NaSCN at 200 mM. (D) R2 of WW4 in the absence (blue) and in the presence of GdmCl at 20 mM (purple) and 200 mM (cyan). (E) R2 of WW4 in the absence (blue) and in the presence of NaSCN at 20 mM (purple) and 200 mM (cyan).
